# Visceral Myopathy Presenting as Acute Appendicitis and Ogilvie Syndrome

**DOI:** 10.1155/2013/906457

**Published:** 2013-04-30

**Authors:** Punyaram Kharbuja, Raghvendra Thakur, Jian Suo

**Affiliations:** Department of Gastrointestinal-Colorectal Surgery, First Hospital of Jilin University, 71th Xin Min Street, Changchun, Jilin 130021, China

## Abstract

*Background*. Visceral myopathy is rare pathological condition of gastrointestinal tract with uncertain clinical presentation and unknown etiology. It often presents with symptoms of chronic intestinal pseudoobstruction of colon. We report a case of visceral myopathy which presented to us as acute appendicitis and Ogilvie syndrome, and we managed it surgically. *Method and Result*. A case report of 20-year female clinically presented as acute appendicitis and we performed laparoscopic exploration which revealed inflamed appendix with grossly dilated ascending colon. We performed laparoscopic appendectomy and postoperatively managed the patients with IV fluids, antibiotics, neostigmine, and extended length rectal tube for enema and decompression. During postoperative period, she developed abdomen distension and peritonitis, and we ordered abdomen CT which revealed colon pseudo- obstruction. We performed right hemicolectomy with permanent ileostomy, and the histopathology reports of resected colon were visceral myopathy. *Conclusion*. Visceral myopathy is very rare group of disease and poorly understood condition that may present with chronic or acute intestinal pseudo-obstruction and often mimic other more common gastrointestinal disease. VM should be considered as differential diagnosis whenever the patient presents with acute appendicitis, uncharacteristic abdominal symptoms, recurrent attacks of abdominal distention, and pain with no radiological evidence of intestinal obstruction.

## 1. Introduction

Visceral myopathy (VM) is rare pathologic conditions of gastrointestinal tract that can mimic various causes of acute abdomen. Patients with VM often present with symptoms of chronic intestinal pseudo-obstruction (CIP) of colon like recurrent episodes of abdominal pain, vomiting, and abdominal distension due to recurrent disturbance of motility in gastrointestinal tract and often causes misdiagnosis of acute abdomen including acute appendicitis. Unlike the classical symptoms, we report a rare and atypical case of VM presenting as acute appendicitis and acute pseudo-obstruction and perforation of colon clinically and pathologically.

## 2. Case Report

20 years, female with 2 days of abdominal pain, pain migrating to right iliac fossa and feverish with temperature 38°C. She denied history of abdominal and gynecological disease or symptoms. Clinically diagnosed acute appendicitis (Alvarado Score 7) and we performed diagnostic laparoscopy which revealed inflamed appendix and grossly dilated ascending colon. As the patient had no history and signs of constipation and intestinal obstruction, we performed only laparoscopic appendectomy. Postoperatively, we managed by intravenous fluids, antibiotics, neostigmine, and extended length rectal tube for enema and decompression. 

She developed abdomen distension, fever, and peritonitis and CT scan showed dilated ascending colon with gas and fecal contains and comparatively thickened colonic wall (Figure 3). After conservative management failed, we did exploratory laparotomy, which revealed marked dilation of ascending colon with serosa tear and perforation. We performed right hemicolectomy with bypass ileostomy and thoroughly searched for any intraabdominal tumor, but we did not find anything that could explain the cause of her intestinal obstruction. Postoperatively we managed conservatively and discharged her on the fifth postoperative day. She has been uneventful for 6 months of followup.

## 3. Pathology Results

Pathology reports based on the full thickness biopsies obtained from operative specimen showed distended colon with 2 mm and less thickness of colon wall.

**Figure 1:**
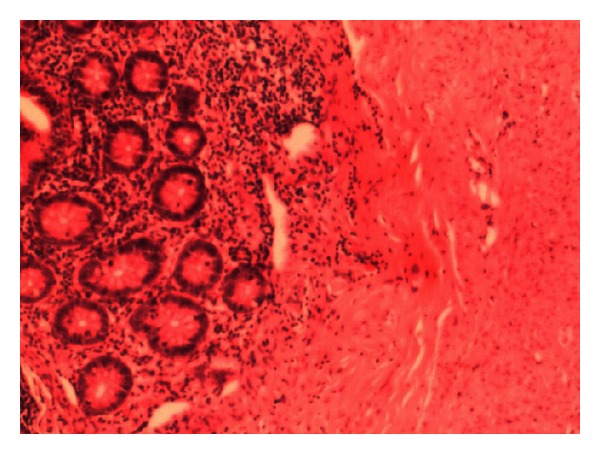
Pathological biopsy specimen shows acutely inflamed appendix.

**Figure 2:**
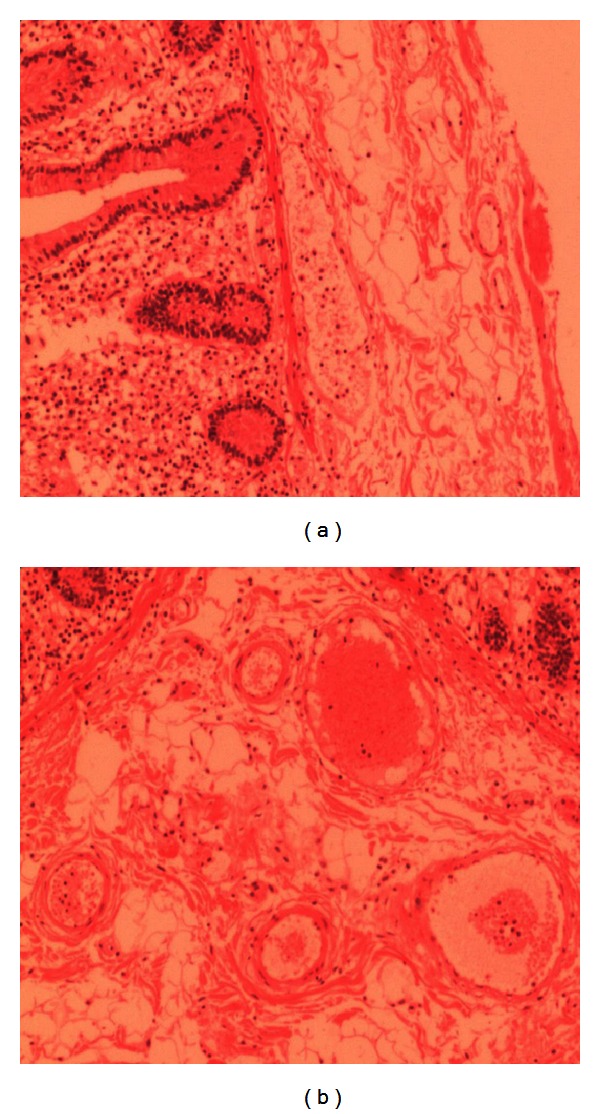
Muscle layer shows vacuolar degeneration, abrupt muscle fiber, and fibrosis, concluding it to be myopathy.

**Figure 3:**
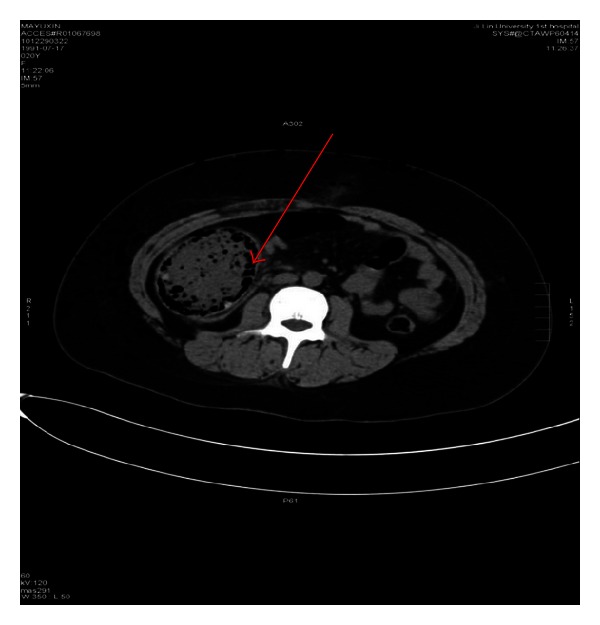
CT scan of abdomen shows distended ascending colon, contained fecal matter, and air entrapment (arrow) with postappendectomy changes.

1. The appendix was inflamed (Figure 1).
2. The mucosa layer was thin and completely disappeared at some points. Submucosa layer was edematous and hemorrhagic. The muscle layer showed vacuolar degeneration and muscle fibers showed abruption, neutrophilic inflammation, mucous degeneration, and fibrosis. Serosa layer was vasodilated with local hemorrhage. This concluded to be final diagnosis of visceral myopathy (Figure 2).

## 4. Discussion

Visceral myopathy is one of the rare diseases that causes intestinal pseudo-obstruction that resulted from neurogenic or myogenic mediated disturbances of gastrointestinal tract motility and failure in propulsive peristalsis [1]. The pathology may be focal, affecting a localized segment of bowel or diffuse, affecting the entire GI tract [2]. VMs are characterized by degeneration and progressive fibrous replacement of intestinal smooth muscles, which result in poor neuromuscular control with subsequent motility disturbances [3]. It may be primary (familial and nonfamilial) or secondary to the other diseases (scleroderma, systemic lupus, stroke, and encephalitis) [4, 5]. Pseudo-obstruction of colon can be acute type Ogilvie syndrome [6] or chronic but patients with VM often present with symptoms of chronic intestinal pseudo-obstruction. Ogilvie syndrome is dilation of the cecum and right colon without nonmechanical obstruction and it occurs in patients with critical illnesses, electrolyte imbalances, anticholinergic medication regimens, and recent surgery but its the relation with VM is not reported yet. Moore et al. reported that diagnosis of VM is based on the distinctive clinical presentation, typical radiologic findings of pseudo-obstruction, and the characteristic morphologic, immunohistochemical, and EM findings seen on the full thickness of intestinal biopsies. Treatment of VM is symptomatic with IV fluids, nutrition, and gastric and colonic decompression by nasogastric and rectal tubes [7]. Surgical resections and enterostomies are required if conservative treatment fails. Surgical resections of nonlocalized VM often have been disappointing results, since the bowel affection will expand further than the resected specimen [8]. In this case right hemicolectomy with permanent right ileostomy was the best option as there was serosa layer tear and perforation on ascending colon which we suppose was due to VM rather than constipation.

VM is poorly understood condition that often presents as chronic intestinal pseudo-obstruction with slow progression of intestinal failure. In this case the presentation was different as acute progression, acute appendicitis, and acute intestinal pseudo-obstruction, and the patient was well nourished. The relation between acute appendicitis and VM and the etiology of VM is still unknown and further study is needed. Ogilvie syndrome and bowel perforation without any prior symptoms are a very atypical presentation of VM in this case.

## 5. Conclusion

VM should be considered as differential diagnosis whenever the patient presents with acute appendicitis, uncharacteristic abdominal symptoms, recurrent attacks of abdominal distention, and pain with no radiological evidence of intestinal obstruction.
